# Correction: GOTHiC, a probabilistic model to resolve complex biases and to identify real interactions in Hi-C data

**DOI:** 10.1371/journal.pone.0177280

**Published:** 2017-05-03

**Authors:** The PLOS ONE Staff

The Funding information appears incorrectly in the published article. The correct Funding information is as follows: This work was supported by the EU, FP7 Epigenesys Network of Excellence, the Francis Crick Institute which receives its core funding from Cancer Research UK (FC001110), the UK Medical Research Council (FC001110), and the Wellcome Trust (FC001110) (NML), the Biotechnology and Biological Science Research Council, UK, the Medical Research Council, UK (PF). BM holds an MRC eMedLab Medical Bioinformatics Career Development Fellowship, funded from award MR/L016311/1”. NML is a Winton Senior Group Leader in recognition of the Winton Charitable Foundation’s support of the establishment of The Francis Crick Institute.

The publisher apologizes for the error.
