# Supplementary figures and images for: GOTHiC, a probabilistic model to resolve complex biases and to identify real interactions in Hi-C data
Source: PLoS One. 2017 Apr 5;12(4):e0174744. doi: 10.1371/journal.pone.0174744 (PMC5381888; doi:10.1371/journal.pone.0174744)

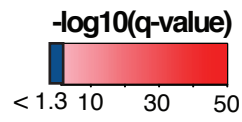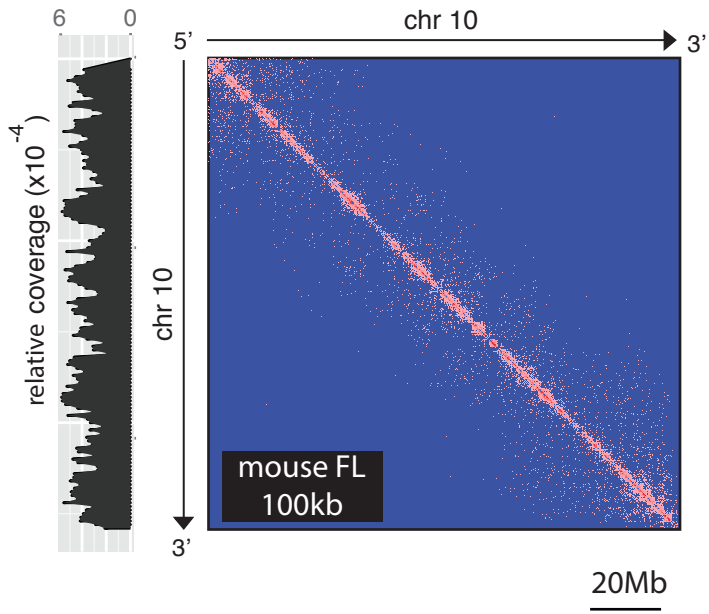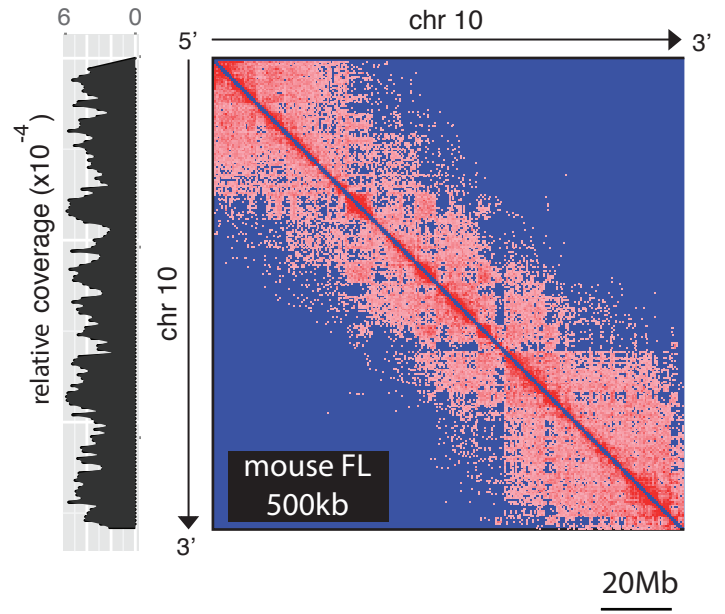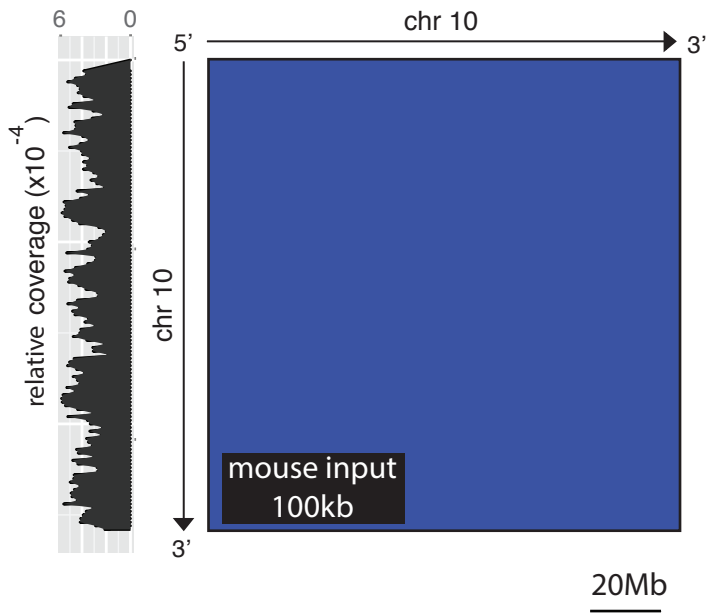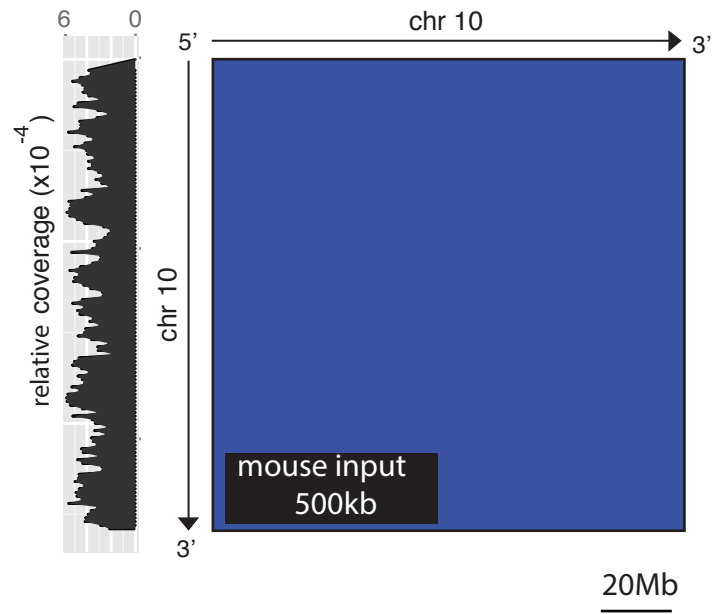

Supplement: S1 Fig — Contact maps of mouse Chromosome 10 representing binomial significances resulting from Hi-C experiment (upper panels) and random ligation experiment (lower panels) in fetal liver cells. Significant interactions are colored with a red gradient as on top. (PDF) [file pone.0174744.s001.pdf]

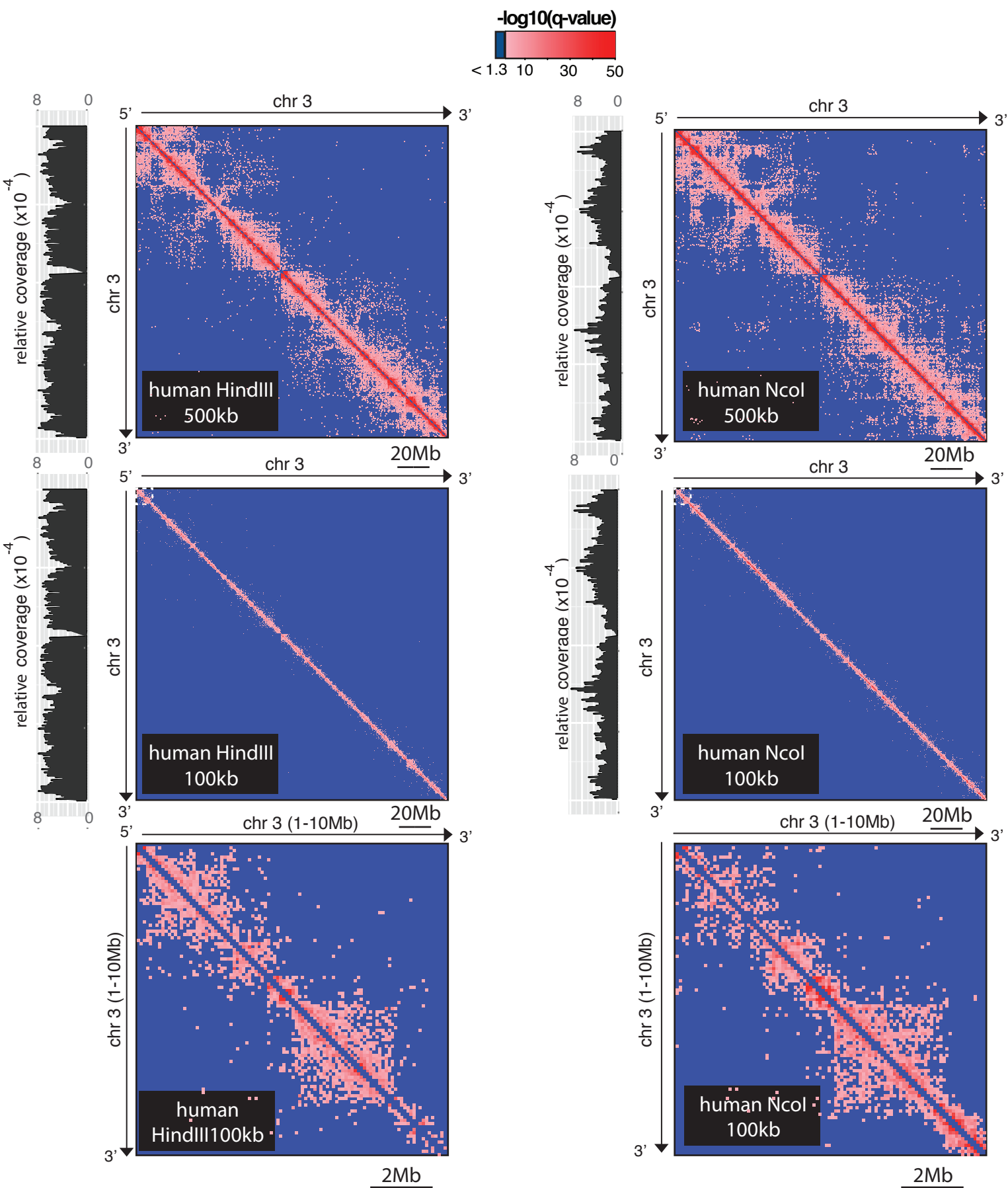

Supplement: S2 Fig — Contact maps of human Chromosome 3 containing raw read counts representing binomial significances resulting from HindIII Hi-C experiment (left panels) and NcoI Hi-C experiment (right panels) at 500kb resolution (upper panels), 100kb resolution (middle and lower panels). The lower panels show a zoom in to chr3 1-10Mb. Significant interactions are colored with a red gradient as on top. (PDF) [file pone.0174744.s002.pdf]

# Human lymphoblastoid

HindIII

NcoI

## A. hiclib

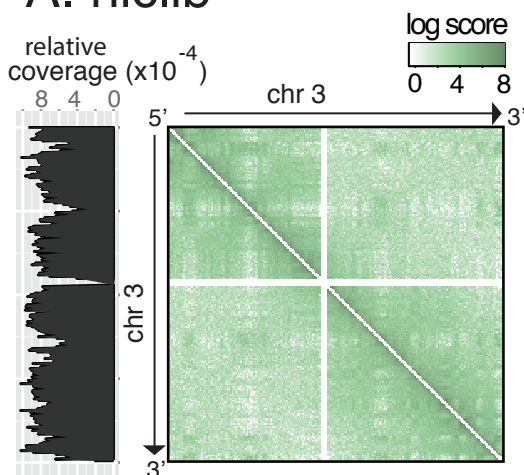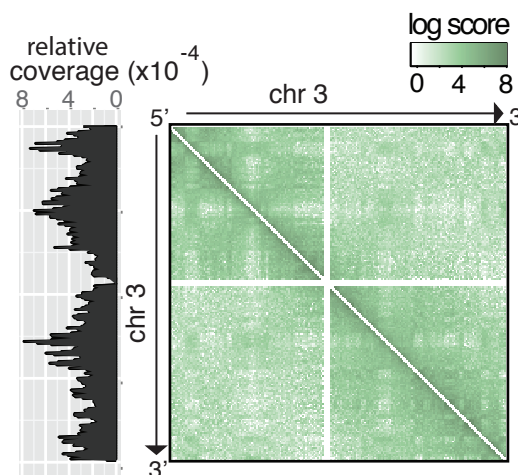

## B. hicpipe

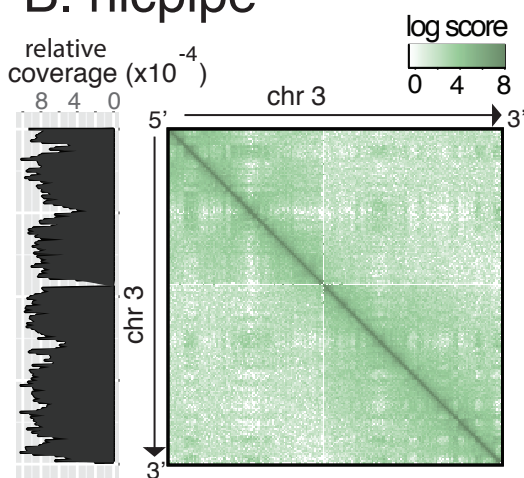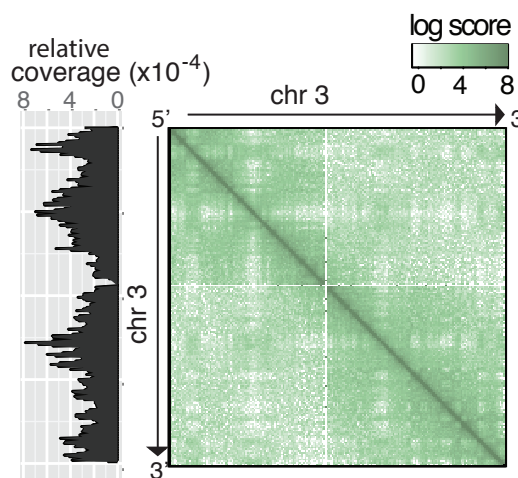

## C

HindIII

NcoI

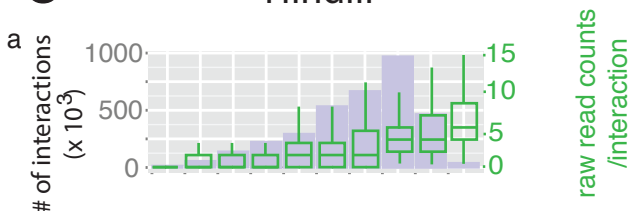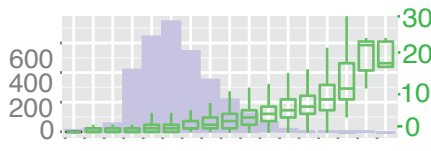

b

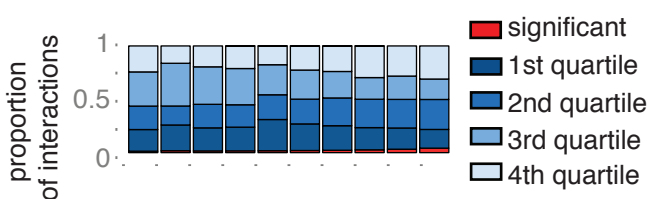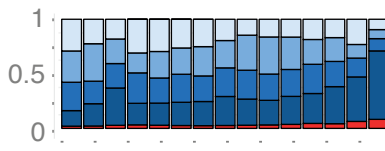

c

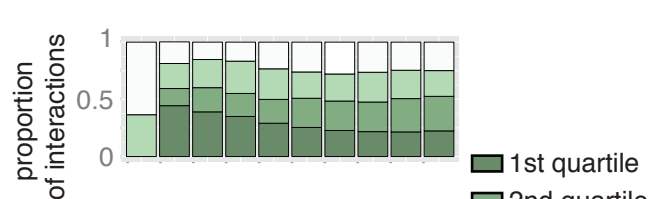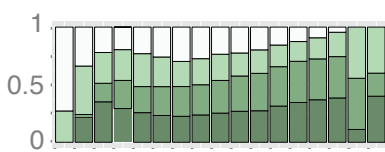

d

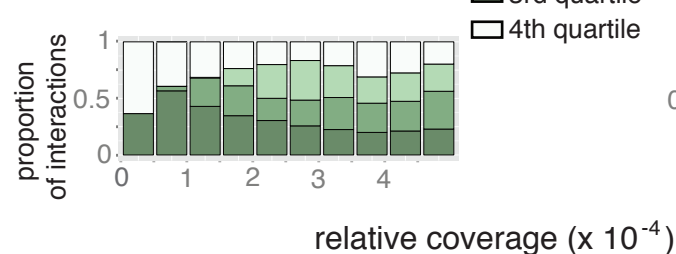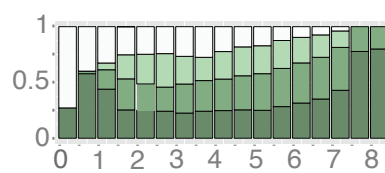

## D

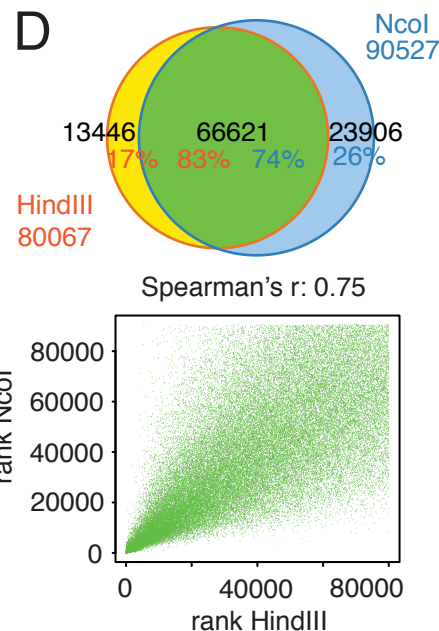

## E

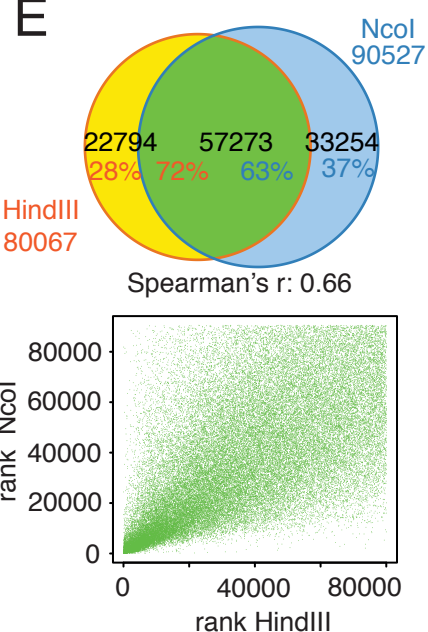

## F

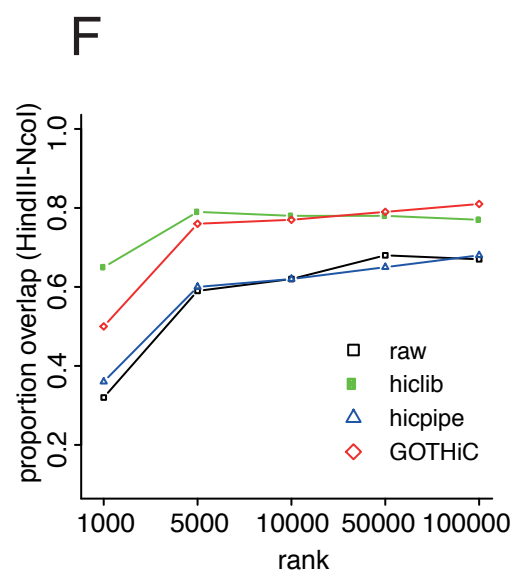

Supplement: S3 Fig — (A-B) Contact maps of human Chromosome 3 containing relative probability computed by hiclib and observed/expected log ratio obtained with hicpipe respectively resulting from HindIII experiment (left panel) and NcoI experiment (right panel). The intensity of the signal is summarized by the gradient above each contact map. (C) Influence of the relative coverage on the distribution of (a) number of observed interactions, (b) GOTHiC, (c) hiclib and (d) hicpipe interaction ranking in the HindIII (left) and NcoI (right) samples. The ranked lists were divided into quartiles, the first quartiles correspond to the top ranked interactions. The distribution of the number of reads per interaction is represented in the top panel with green box plots (corresponding y-axis is placed on the right of the plot). 80,067and 90,527 interactions were called significant using GOTHiC in the HindIII and NcoI samples respectively. In order to compare with the predictions of (D) hiclib and (E) hicpipe, we selected the 80,067 and 90,527 top ranked interactions of these methods and computed the overlap (top) and correlation (bottom) between the two samples. (F) Proportion of overlap between HindIII and NcoI samples by ranking of interactions according to raw read counts (black), relative probability computed by hiclib (green), observed/expected log ratio obtained with hicpipe (blue), siqnificance by GOTHiC (red). (PDF) [file pone.0174744.s003.pdf]

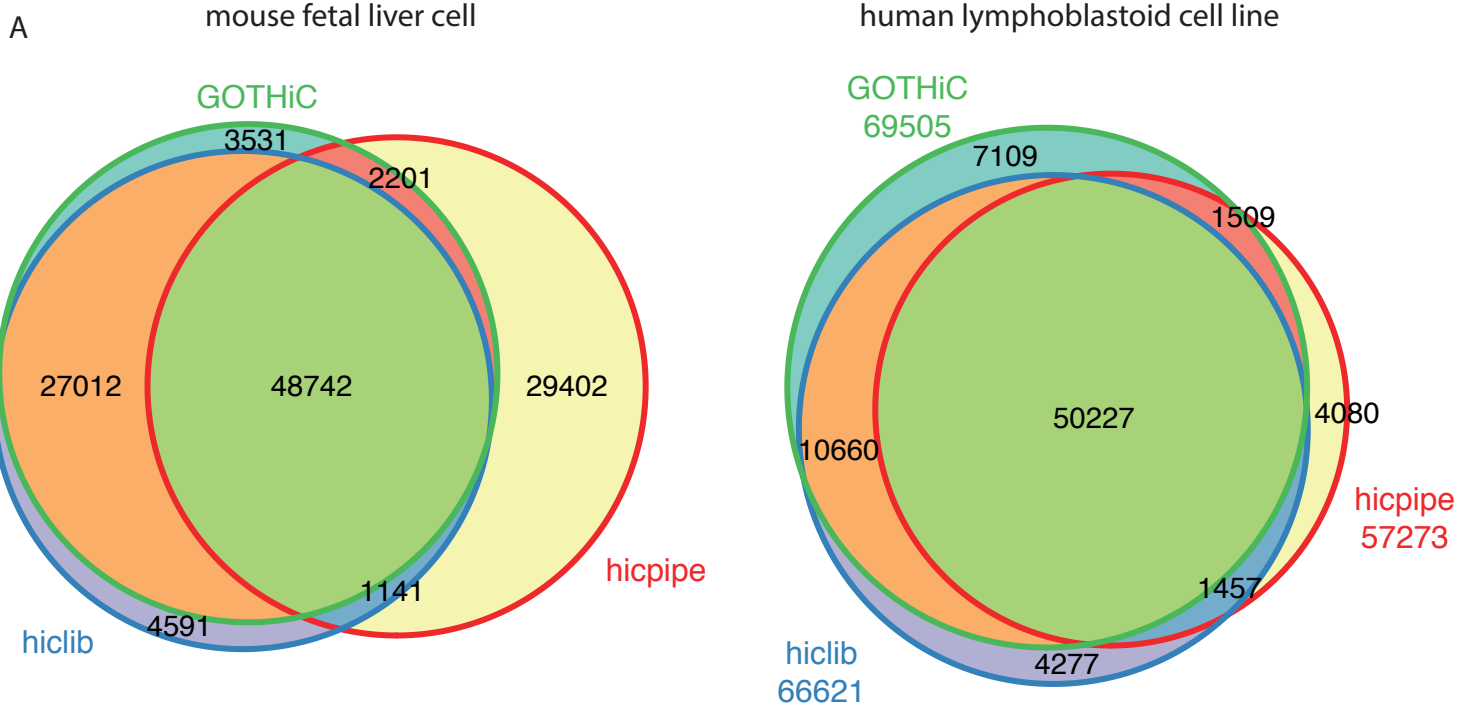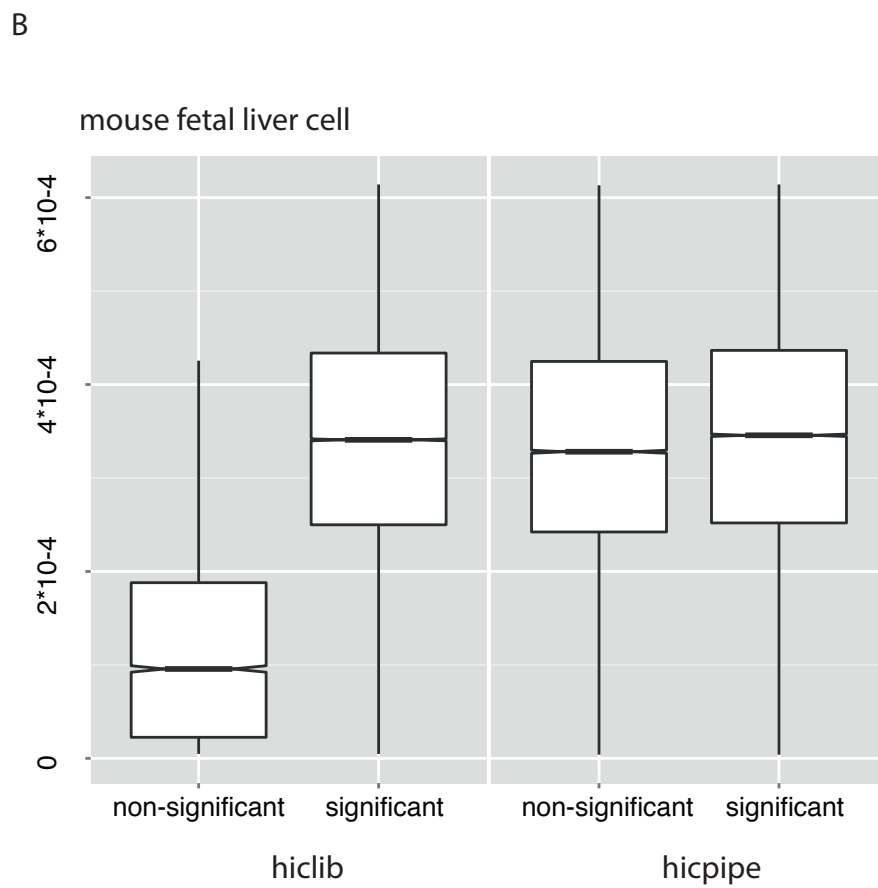

Supplement: S4 Fig — GOTHiC identified 80,085 significant interactions in the mouse fetal liver cell Hi-C dataset. (A) Venn diagram showing the overlap between the significant interactions identified by GOTHiC and the top 80,085 interactions from the hiclib and hicpipe outputs. (B) There were 69505 significant interactions detected by GOTHiC that overlapped between the HindIII and NcoI experiments in the human lymphoblastoid cell line, 57,273 by hicpipe and 66,621 by hiclib. The Venn diagram shows the overlap between the GOTHiC, hiclib and hicpipe overlapping interactions. (PDF) [file pone.0174744.s004.pdf]
